# Influence of Environmental Stressors on the Microbiota of Zebra Mussels (*Dreissena polymorpha*)

**DOI:** 10.1007/s00248-020-01642-2

**Published:** 2020-11-26

**Authors:** Prince P. Mathai, Jonathan H. Bertram, Soumesh K. Padhi, Vikash Singh, Isaiah E. Tolo, Alexander Primus, Sunil K. Mor, Nicholas B. D. Phelps, Michael J. Sadowsky

**Affiliations:** 1 BioTechnology Institute, University of Minnesota, 1479 Gortner Ave., 140 Gortner Labs, St. Paul, MN 55108 USA; 2 Department of Fisheries, Wildlife, and Conservation Biology, University of Minnesota, St. Paul, MN USA; 3 Department of Veterinary Population Medicine, University of Minnesota, St. Paul, MN USA; 4 Department of Soil, Water, and Climate, University of Minnesota, St. Paul, MN USA; 5 Department of Plant and Microbial Biology, University of Minnesota, St. Paul, MN USA

**Keywords:** Zebra mussels, Stress, Temperature, Salinity, Microbial communities, Pathogens, Invasive species

## Abstract

Host-associated microbiota play a critical role in host fitness by providing nutrition, enhancing digestion capabilities, and by providing protection from pathogens. Here, we investigated the effects of two environmental stressors, temperature, and salinity, on the microbiota associated with zebra mussels (ZMs), a highly invasive bivalve in North America. To examine this in detail, lake-collected ZMs were acclimated to laboratory conditions, and subjected to temperature and salinity stress conditions. The impact of these stressors on the diversity, composition, and dynamics of ZM-associated microbiota were assessed by using amplicon- and shotgun-based sequencing, and qPCR-based approaches. Elevated temperature was found to be the primary driver of ZM mortality, although salinity alone also increased its likelihood. Stressor-induced ZM mortality, which ranged between 53 and 100%, was concomitant with significant increases in the relative abundance of several genera of putative opportunistic pathogens including *Aeromonas*. These genera were only present in low relative abundance in ZMs obtained from the control tank with 0% mortality. Shotgun sequencing and qPCR analyses indicated that the relative and absolute abundances of pathogenic *Aeromonas* species (particularly *A. veronii*) were significantly greater in temperature-induced dead ZMs. Taken together, our results show that environmental stress, especially elevated temperature (> 25 °C), is associated with the rapid mortality of ZMs as well as the proliferation of putative opportunistic bacterial pathogens.

**Supplementary Information:**

The online version contains supplementary material available at 10.1007/s00248-020-01642-2.

## Introduction

The dreissenid *Dreissena polymorpha*, commonly known as the zebra mussel (ZM), is among the most successful aquatic animals in the Anthropocene. These bivalve mollusks, native to the Caspian Sea, were introduced to Lake Erie (USA) in the 1980s, most likely via ballast water discharged from transatlantic shipping vessels [1]. Since this time, these invasive suspension-feeders have proliferated in thousands of waterbodies in the Northeastern and Midwestern USA [2]. Strategies to contain the spread and reduce the resultant damage due to ZM proliferation have generally been unsuccessful. Thus far, ZMs have been discovered as far south as Texas and as far west as California, progressively expanding into new territories and climate regimes [3, 4]. The pervasiveness, behavior, and resiliency of ZMs have secured them a role as an “ecological engineer” in many aquatic ecosystems [5].

ZMs have several physiological traits that contribute to their success as an invasive species, including a high suspension-feeding capacity, high fecundity, and an affinity for hard substrates such as shells of native mussel species [6, 7]. ZMs alter trophic systems by removal of plankton from the water column and deposition on the bottom of the waterbody [8]. Associated direct ecosystem changes include a decrease in zooplankton and phytoplankton, an increase in benthic primary productivity, and an increase in benthic microbial biodiversity [9–12]. ZMs indirectly affect piscivorous fish populations and increase light penetration, thus increasing the littoral zone of a waterbody and enhancing the growth of attached benthic macrophytic algae [13].

Despite successful establishment in some systems, introduced bivalves, like ZMs, are susceptible to environmental extremes and climate variation -conditions many native species are able to endure [14, 15]. For instance, over 99% of an invasive Asian clam (*Corbicula fluminea*) was extirpated from a river in Southern USA because of drought [16]. A few studies have addressed the impact of environmental perturbations on ZMs, specifically that they suffer physiological stress and acute mortality at temperatures greater than 30–33 °C [17, 18] and prefer salinities less than 4 ppt [19, 20]. Mollusks are both poikilothermic and ectothermic, and thus their body temperature is variable and dependent on the water in which they reside. Both absolute temperatures and abrupt temperature changes can significantly impact rates of metabolism, immunological function, and disease susceptibility. Like other bivalves, ZMs harbor a large number of microbiota [21], some of which are postulated to play major roles in their growth and survival. It seems likely that changes in environmental factors (e.g., temperature) would also impact the composition of microbial communities associated with such organisms, either directly or indirectly. Therefore, given the pivotal role of ZMs in aquatic ecosystems and in light of changing climate regimes, it is crucial to understand the relationships between ZMs and how their associated microbiota respond to stressors, and environmental perturbations. This information may also be useful for future biocontrol efforts.

The objective of this study was to understand how ZMs and their associated microbiota respond to temperature and salinity stress. We hypothesized that these stressors would alter the composition of ZM-associated microbial communities potentially leading to the outgrowth of adapted opportunistic pathogens which induce ZM mortality. To test this hypothesis, ZMs were harvested from a natural lake environment, acclimated to laboratory conditions in aquaria, and subjected to temperature and salinity stress conditions over a period of 6 weeks. The impact of these stressors on ZM-associated microbial communities was examined by using a combination of amplicon- and shotgun-based sequencing, and via quantitative PCR-based approaches.

## Methods

### Field Sampling

Prior to commencing this study, a permit was obtained from the Minnesota Department of Natural Resources (MNDNR) to collect, possess, and transport ZMs from lakes with established dreissenid populations. Over 2,500 ZMs were collected on September 5, 2018 within a 30-m^2^ area of Gull Lake in Minnesota (USA) (GPS coordinates: 46.3963529, − 94.3686372). Mussels (1.8 to 3.5-cm length) were collected within depths of 0.2–1.0 m from hard surfaces including stones, rocks, and clusters of mussels (druses).

Water temperature, pH, and dissolved oxygen concentrations were measured at time of collection using a handheld thermometer (Taylor; Oak Brook, IL, USA), pH meter (Vantakool; Elizabeth, CO, USA), and a MW 600 Smart DO meter (Milwaukee Instruments; Milwaukee, WI, USA), respectively. At the time of ZM collection, the lake water temperature was 21.7 ± 0.6 °C, had a pH of 8.86 ± 0.04 and dissolved oxygen concentration was 7.2 ± 0.2 mg/L. The ZMs were kept in lake water in sterile 1 L plastic containers (Nalgene; Rochester, NY, USA) and transported on ice to the invasive species containment facility (Minnesota Aquatic Invasive Species Research Center; [MAISRC], University of Minnesota; Saint Paul, MN), within 24 h of collection.

### Tank Setup and Maintenance

All ZM tank experiments were conducted in a biosafety level-2 lab located within the MAISRC containment facility at the University of Mnnesota. The ZMs were initially transferred to a holding tank (110 L capacity, 30 cm × 45 cm × 85 cm) containing a 4:1 mixture of well water and filtered lake water, sourced from the sampling site. After 24 h, the ZMs were divided into six 110-L tanks (A–F; 5 experimental tanks and 1 control). During tank division, deceased, and visibly moribund ZMs were removed. Individual ZM counts ranged between 189 and 260 per tank, and ZMs were subdivided in glass Petri dishes (90-mm diameter × 15-mm height) into groups of approximately 20 individuals. Each experimental tank contained a 4:1 mixture of well water and pooled lake water, kept at 17 ± 1 °C. Lake water was pooled from four lakes (Gull–Crow Wing County, Como–Ramsey County, and Harriet and Bde Maka Ska–Hennepin County) to obtain diverse bacterioplankton communities, and filtered through sterile double cheese cloth to remove large debris. Each tank was gently aerated, maintained in an ambient room temperature of 17 ± 1 °C and illuminated under a photoperiod of 12 h light: 12 h dark cycle. ZMs were fed every day with 5-ml PhytōChrōm (approximately 200 million phytoplankton cells per mL, particle size: 1–30 μm, Brightwell Aquatics; Fort Payne, AL, USA) per 110 L of tank water, as per labeling instructions. The feed comprised six species of marine algae, which included *Haematococcus* sp., *Nannochloroposis* sp., *Tetraselmis* sp., *Isochrysis* sp., *Pavlova* sp., and *Thalassiosira* species. We postulated that the wide range of food particle size (1–30 μm) would be more reflective of a natural habitat, allowing both selection and rejection of particles during ZM filtration feeding.

Water chemistry (temperature, pH, and DO) were monitored daily throughout the course of this experiment (Fig. S1), as described previously. In addition, salinity and total ammonia nitrogen (TAN) concentrations were measured daily, with a portable salinity refractometer (Agriculture Solutions; Strong, ME, USA) and an ammonia test kit (API; Chalfont, PA, USA), respectively. Every 2–3 days, approximately 10% of the tank water was discarded and replaced with fresh well water, so as to maintain TAN concentrations below 0.25 mg/L.

During the acclimation period, infirm, and deceased ZMs, identified by visual cues and motor response, were removed. The health status of the ZMs was assessed by disturbing the water near an individual ZM. Healthy ZMs would quickly retract their siphons and close their shells upon disturbance. Infirm ZMs that were found to suffer mortality shortly thereafter exhibited a slowed response, and deceased ZMs exhibited no motor response. The acclimation phase lasted for 14 days (temperature 17 ± 1 °C; 12 h light: dark cycle; with daily feeding regiment), after which experimental stressors were initiated in 5 tanks (A, B, C, D, and E).

### Application of Stressors in Experimental Tanks

After the acclimation phase, the stressors (temperature and salinity) were slowly increased during the ramp-up phase, which lasted between 10 and 14 days, depending on the tank. The temperature stressor limits (medium temp: 26 °C and high temp: 32 °C) were selected based on sublethal and lethal temperature-induced mortality limits determined in prior studies [22]. A salinity stressor limit of 13.5 ppt was selected based on the range of saline habitats encountered by ZMs in North America and those of ancestral habitats [18, 23]. Moreover, since environments supporting the growth of ZMs are dynamic, an alternating temperature scenario method was imparted to compare temperature accumulation damage and provide a secondary signal for daily circadian rhythms.

The water temperature in tanks A, B, D, and E, was increased at approximately 1 °C per day using two 200-W digital aquarium heaters per tank (Aquatop; Brea, CA, USA), whereas the salinity in tanks C and D was increased by approximately 2 ppt every 2 days by adding an aquarium salt formulation (Instant Ocean; Blacksburg, VA, USA). After reaching the final set point (tank A: 32 °C, 1.5 ppt; tank B: 32 °C, 13.5 ppt; tank C: 17 °C, 13.5 ppt; tank D: 32 °C, 1.5 ppt; tank E: 26 °C, 1.5 ppt), constant conditions in all tanks were maintained, except for tank A where heat was provided for only 16 h each day. These conditions were maintained for 28 days or until the conclusion of the experiment. Tank F (control) was maintained at room temperature (17 ± 1 °C), with no salinity added (1.5 ppt), throughout the duration of this experiment.

### Sample Collection and Processing

ZMs were periodically collected for microbial community analysis by removing at least 5 randomly selected, visibly healthy, “live” individuals from each tank per timepoint (Fig. S2). In addition, dead ZMs, identified by visual cues and lack of motor response, were collected during daily monitoring, to limit the growth of saprophytic bacteria in dead or dying ZMs. Upon collection, all ZMs were immediately stored at − 80 °C for later processing. Prior to processing, the frozen ZMs were thawed to room temperature, and the exterior shell of each individual was gently scrubbed with 70% ethanol using a toothbrush. The shell was opened by making an incision to the adductor muscle using a sterile razor blade. The internal tissue was aseptically transferred to a 2-ml microcentrifuge tube containing 500 μl of 1x phosphate buffered saline (pH 7.0) and vortexed for 1 min. The tissue was homogenized with a sterile glass tissue grinder for 1 min and tubes were vortexed for an additional 5 min. The homogenate was centrifuged at low speed (500×*g*) for 10 min to separate larger cell debris from the supernatant. The resulting supernatant was stored at − 80 °C until used.

Water samples (100 ml) were collected from each tank (total = 23; sampling timeline depicted in Fig. S5) and filtered through 5-μm mixed cellulose filters (MF-Millipore; Darmstadt, Germany), followed by 0.22-μm filters (MF-Millipore). Both the MF filters were pooled together and transferred to a 15-ml tube and 4 ml of 0.01% sodium pyrophosphate buffer, pH 7.0, containing 0.2% Tween 20, was added. Tubes were vortexed for 3 min, centrifuged at 13,000×*g* for 5 min, and the pellets were stored at − 20 °C.

DNA was extracted from thawed frozen supernatant of ZM tissues and cell pellets of water samples using the DNeasy PowerSoil Kit (Qiagen; Hilden, Germany) as per manufacturer’s instructions. DNA concentrations were measured using Qubit 2.0 Fluorometer (ThermoFisher Scientific; Waltham; MA, USA). The extracted DNA samples were stored at -20 °C until further analysis.

### Amplicon Sequencing, Bioinformatics, and Statistical Analysis

DNA samples (*n* = 405) were sequenced at the University of Minnesota Genomics Center (UMGC; Minneapolis, MN) by using universal primers: 515f (5’-GTGCCAGCMGCCGCGGTAA-3’) and 806r (5’-GGACTACHVGGGTWTCTAAT-3’) targeting the V4 region of the 16S rRNA gene as described elsewhere [24]. Bar-coded sequencing was performed on the MiSeq platform (Illumina, San Diego, CA) using a 2 × 300-bp paired end protocol. All fastq files were deposited in the NCBI Sequence Read Archive under BioProject accession number PRJNA645687.

Sequences were analyzed using QIIME v.1.8.0 [25]. Illumina adapters and low quality regions (< Q30) were removed using Trimmomatic v. 3.2 [26]. Reads with less than 75% of the total amplicon length were discarded and high-quality reads were joined in pandaseq using the fastqjoin script [27, 28]. Chimeras were identified using UCHIME v. 6.1 [29]. A naïve Bayesian classifier was used to classify sequences against the RDP training set v. 9 at 80% bootstrap confidence score [30]. Open-reference operational taxonomic units (OTUs) were clustered at 3% dissimilarity using UCLUST and compared against the SILVA v.132 16S rRNA database using PyNast [31–33]. OTU counts were rarefied to 10,000 sequences per sample for statistical analysis.

Alpha diversity measures were calculated using Good’s coverage, observed OTUs, Chao1, Shannon’s H and Simpson’s E indices. Bray–Curtis dissimilarity matrices were used for principal coordinate analysis (PCoA). These matrices were also used to assess statistical significance of beta diversity between different ZM groups by using permutational multivariate analysis of variance (PERMANOVA) [34] using the Adonis function in the R package vegan [35] with 999 permutations. The PERMDISP function was used to examine whether the dispersions between groups were significant [36]. Redundancy analysis (RDA) was performed to determine which taxa best explained ZM mortality using XLSTAT Ecology v 19.6 (Addinsoft; New York, NY USA). In addition, taxa that were overrepresented in dead ZMs (tanks A–E) were identified (vs. control tank F) using linear discriminant analysis effect size (LEfSe) analysis [37]. A *p* value less than 0.05 was considered to indicate statistical significance for all tests.

### Shotgun Metagenomic Sequencing and Analysis

A total of 28-pooled ZM tissue samples (10 individuals per timepoint) were obtained from the six tanks and processed for shotgun sequencing as described above. The frozen homogenates (stored in − 80 °C) were freeze-thawed three times, and centrifuged at 2,896×*g* for 25 min at 4 °C. The supernatant was treated with TURBO™ DNase (Ambion; Austin, TX, USA) and RNase A (ThermoFisher Scientific) for 1 h at 37 °C to reduce host nucleic acid interference. Nucleic acids were extracted using QIAamp MinElute Virus Spin Kit (Qiagen) by following manufacturer’s instruction with slight modification. Linear acrylamide was used as a carrier of nucleic acids instead of carrier RNA. The extracted nucleic acids were submitted to the UMGC for library preparation and sequencing. Library preparation was completed using the dual-indexed Stranded Total RNA Pico Mammalian kit, according to manufacturer instructions (Clontech, a Takara Bio Company, CA, USA). Libraries were normalized according to the median fragment size measured by Tape Station 2.0 (Agilent) and library concentration measured by Qubit. Sequencing was performed on the HiSeq 2500 platform (Illumina) using a 2 × 125-bp paired end protocol. After automated cluster generation, the genomic sequence reads (fastq) files were obtained for further analysis. Species-level taxonomic profiling was performed using Kraken, which unambiguously classifies shotgun metagenomic reads based on the analysis of shared k-mers between input reads and pre-compiled database of genomes [38]. All fastq files were deposited in the NCBI Sequence Read Archive under BioProject accession number PRJNA645687.

### Quantitative PCR

The total bacterial 16S rRNA and cytotoxic enterotoxin (*act*) genes in ZM samples were quantified using primer sets: 515f (5’-GTGCCAGCMGCCGCGGTAA-3’) and 806r (5’-GGACTACHVGGGTWTCTAAT-3’) [39], and AHCF (5’-GAGAAGGTGACCACCAAGAACA-3’) and AHCR (5’-AACTGACATCGGCCTTGAACTC-3’) [40], respectively. The qPCRs were done using a StepOnePlus Real-Time PCR System (Applied Biosystems, Foster City, CA, USA). The reaction mixture (12 μl) contained iTaq Universal SYBR Green Supermix (Bio-Rad, Hercules, CA, USA), 500 nM each forward and reverse primer, and 6 μl DNA template. PCR was performed, in duplicate, under the following conditions: initial denaturation at 95 °C for 10 min, followed by 40 cycles of 95 °C for 15 s and either 55 °C (total 16S rRNA gene) or 60 °C (*act* gene) for 1 min. Each qPCR run included DNA standards and no-template controls. Melt-curve analysis was performed after each run to confirm reaction specificity. The threshold cycle value was determined using the StepOnePlus™ Software v2.3 (Applied Biosystems).

## Results

### Stressor-Induced ZM Mortality

**Fig. 1:**
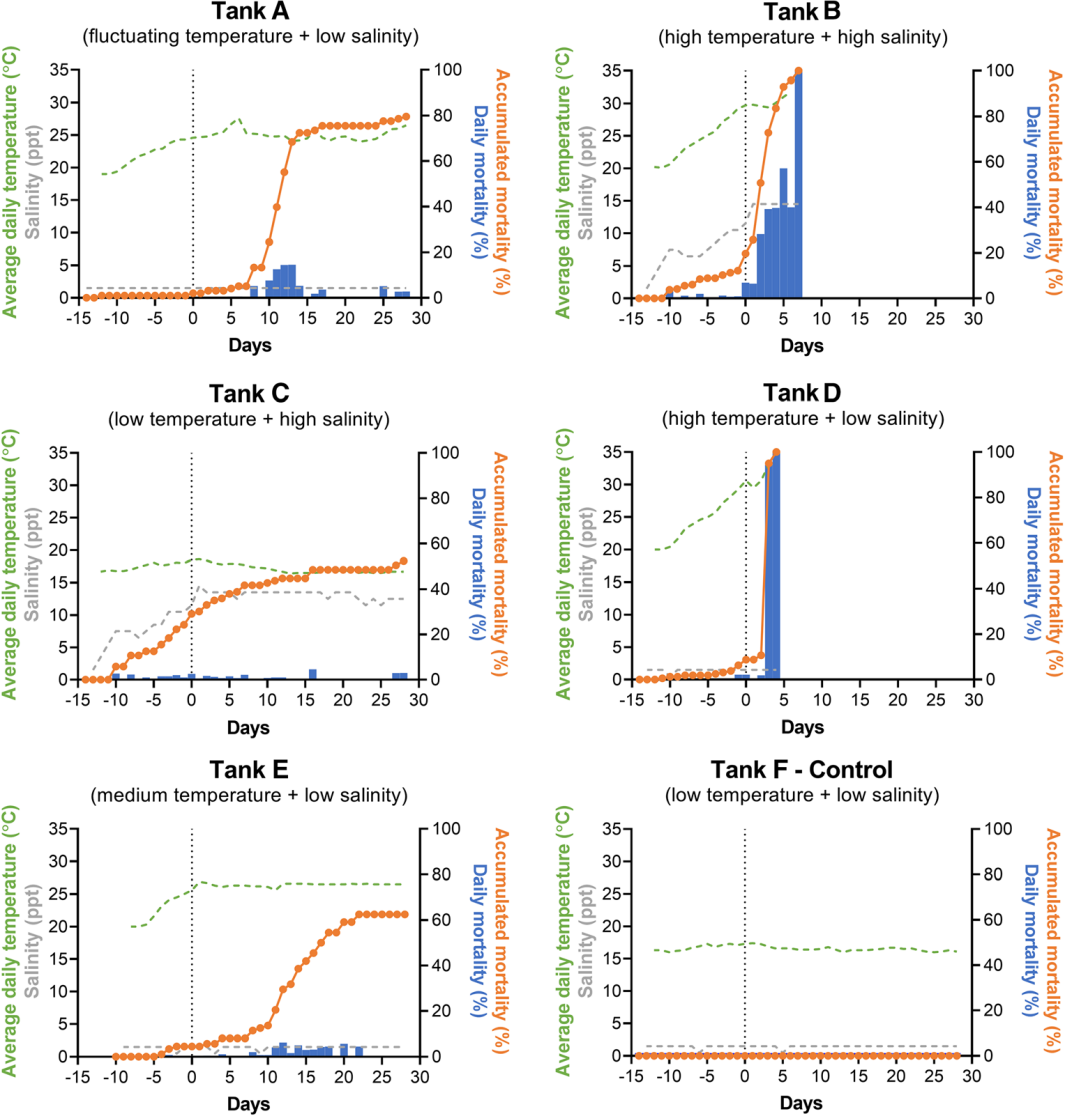
Tank experiments demonstrating the effect of water temperature and salinity on ZM survival. Dashed vertical lines indicate the end of the acclimation phase (14 d) and the start of experimental phase (28 days) in tanks A–E

Elevated temperature was the primary driver of ZM mortality, although salinity increased the likelihood of mortality as well (Fig. 1, Table S1). Heat stress to ZM appeared to accumulate during the acclimation and experimental phase when thermal stress was applied. ZM mortality increased dramatically as the temperatures reached 30 °C in tanks D, B, and A. Based on pre\trial observations, the rate of mortality was considered severe if greater than 2.5% of the tank population was lost daily. The ZMs in tank D (high temp) withstood temperatures greater than 30 °C for 2 days, however 95% were dead by the third day, resulting in early experimental termination. Similarly, over 33% of the ZMs within tank B (high temp and high salinity) suffered mortality within two experimental days of the water temperature surpassing 30 °C, with the remainder being lost by day 7. The fluctuating temperature condition in tank A increased mortality after 10 days (at experimental temp), although the rate of loss decreased after 14 days and remained low for the duration of the experiment. Tank E (medium temp) reached a maximum temperature of 26 °C, and accumulated thermal stress at a much lower rate: after 11 days, the rate of ZM loss increased from 0.5% ± 0.6% ZMs per day to 3.1% ± 2.6% ZMs per day and remained low for the duration of the experiment. While tank C was kept at room temperature (17 ± 1 °C), it had the same salinity level as tank B (13.5 ppt). A steady and low mortality rate (1.2% ± 0.8% per day) began as soon as the salinity was increased, but death rate remained consistent for the first 12 days of the experiment, before dropping to 1.0% ± 1.6% deaths per day for the remaining 16 days. There was no ZM mortality in tank F (control) during the acclimation or experimental phases. The treatments employed were successful in stressing ZMs, and the proportion of dead ZMs in each tank at the end of the experiment were as follows: tank A (fluctuating temp) 79.6%, tank B (high temp and high salinity) 100%, tank C (high salinity) 52.4%, tank D (high temp) 100%, tank E (medium temp) 70.5%, and tank F (control) 0%.

### Effect of Stressors on Microbiota Associated with ZMs

High-throughput DNA sequencing of 405 samples generated approximately 31 million paired-end reads, of which 88% of sequences passed the quality filtration steps. Seven samples were removed from subsequent analyses as they did not meet the minimum cutoff of 10,000 sequences per sample. Sequence analysis found a range of 244 to 1316 OTUs among all samples, with a mean ± standard deviation Good’s coverage of 97.1% ± 1.0%.

When measured by the Shannon index, live ZMs from tank F had significantly greater alpha diversity than did the dead ZMs from experimental tank D (*p* = 0.001) (Table 1). In all the experimental tanks, dead ZMs were less diverse compared to live ZMs (Table 1). A reduction in diversity was also observed in some live ZMs collected from the experimental tanks prior to any mortality events (Fig. S2). Species richness, estimated using the Chao1 index, was significantly different between live ZMs from tank F and dead ZMs from tank E (*p* < 0.0001), and with live ZMs from tank A (*p* = 0.004) (Table 1). However, no significant differences (*p* > 0.05) were observed in Simpson’s evenness between live ZMs in tank F and live/dead ZMs from all other tanks (Table 1).

**Table 1:** Coverage and alpha diversity indices for microbial communities among ZM samples collected during the experimental phase, consolidated by tank and mortality status

Tank	Status	*n*	Good’s coverage (%)	Observed OTUs	Shannon’s H	Chao1	Simpson’s E
A	Live	38	96.7 ± 1.1	759 ± 224 ^A^	5.87 ± 1.61 ^A^	1311 ± 369 ^A^	0.030 ± 0.031 ^A^
	Dead	28	96.6 ± 1.1	616 ± 229 ^ABC^	5.00 ± 1.28 ^ABC^	1307 ± 372 ^A^	0.021 ± 0.013 ^A^
B	Live	20	97.2 ± 0.6	615 ± 99 ^ABC^	5.71 ± 0.45 ^AB^	1187 ± 178 ^AB^	0.024 ± 0.016 ^A^
	Dead	17	97.8 ± 0.4	410 ± 75 ^D^	4.58 ± 1.01 ^BC^	920 ± 175 ^BC^	0.028 ± 0.018 ^A^
C	Live	35	97.3 ± 0.7	566 ± 118 ^BCD^	5.43 ± 0.85 ^AB^	1092 ± 219 ^ABC^	0.031 ± 0.018 ^A^
	Dead	18	97.2 ± 0.8	503 ± 155 ^CD^	4.62 ± 0.95 ^ABC^	1110 ± 296 ^ABC^	0.021 ± 0.014 ^A^
D	Live	10	96.9 ± 0.7	661 ± 82 ^ABC^	5.60 ± 1.21 ^AB^	1266 ± 161 ^A^	0.034 ± 0.031 ^A^
	Dead	11	97.8 ± 0.6	400 ± 99 ^D^	3.95 ± 0.78 ^C^	880 ± 187 ^C^	0.018 ± 0.011 ^A^
E	Live	35	96.7 ± 0.9	684 ± 155 ^AB^	5.54 ± 1.42 ^AB^	1240 ± 293 ^A^	0.031 ± 0.030 ^A^
	Dead	30	96.4 ± 1.3	654 ± 254 ^ABC^	5.11 ± 1.33 ^ABC^	1358 ± 455 ^A^	0.021 ± 0.011 ^A^
F (control)	Live	45	97.4 ± 0.7	609 ± 169 ^BC^	5.71 ± 1.27 ^AB^	1084 ± 209 ^ABC^	0.038 ± 0.029 ^A^

Values are means ± standard deviations among all samples

Sample groups sharing the same letter did not differ significantly in alpha diversity by Tukey’s post hoc test (*p* > 0.05)

**Fig. 2:**
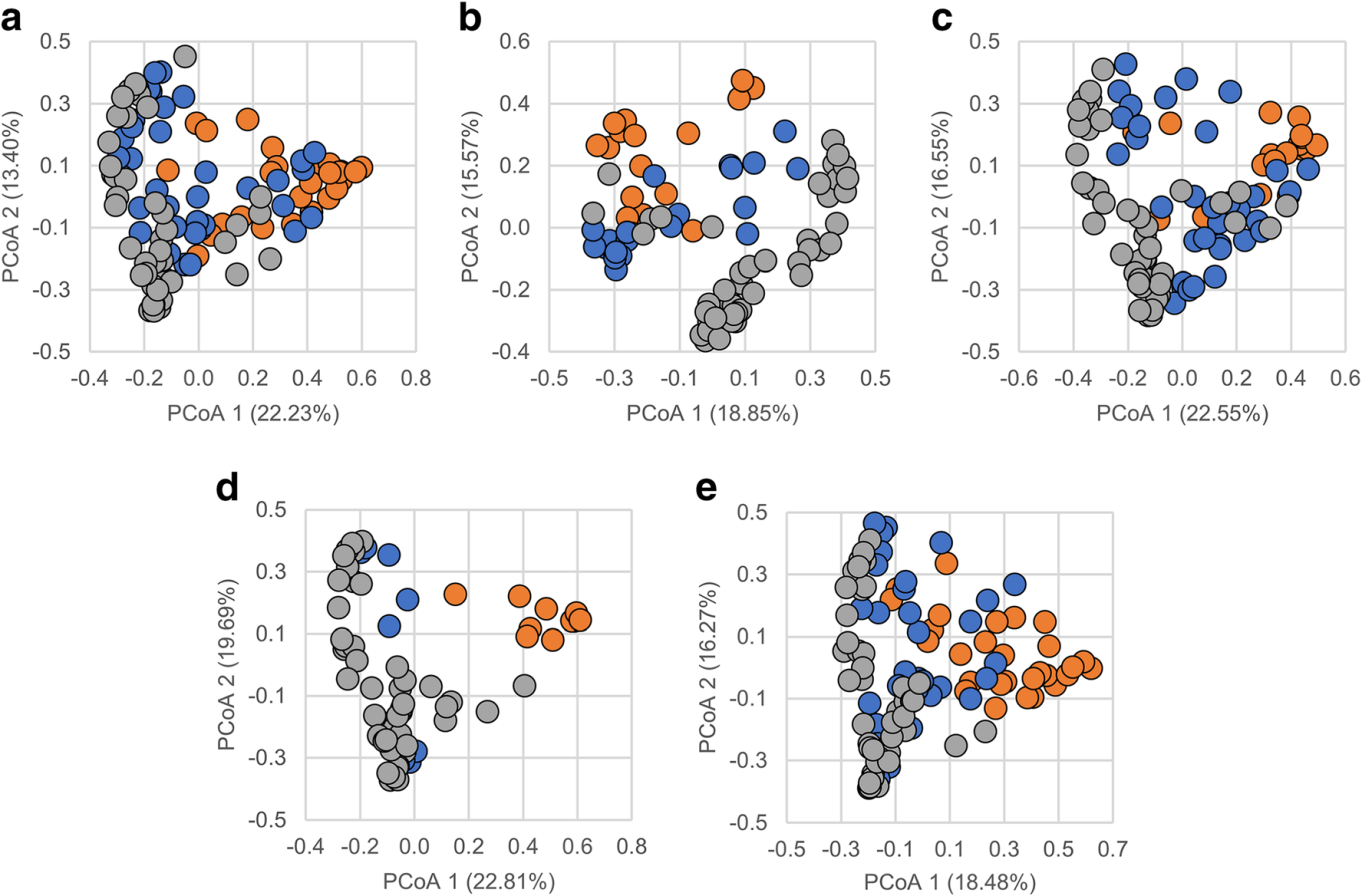
Principal coordinates analysis of microbial communities associated with live and dead ZMs in each experimental tank (A–E). Live ZMs from the control tank (F) has been included in each plot for visualization purposes. Each circle represents an individual ZM. Legend: Live ZMs from experimental tanks (blue), dead ZMs from experimental tanks (orange), and live ZMs from control tank (grey)

Regardless of the stressor (i.e., temperature or salinity), significant differences among the microbiota from live and dead ZMs in each experimental tank (A–E) and live ZMs from the control tank F were detected with Permanova (*R*^2^ = 0.153 to 0.218, *p* = 0.001) (Table S2). These results were visualized using principal coordinates analysis (Fig. 2), which explained 34.8 to 42.5% of the total variation observed.

**Fig. 3:**
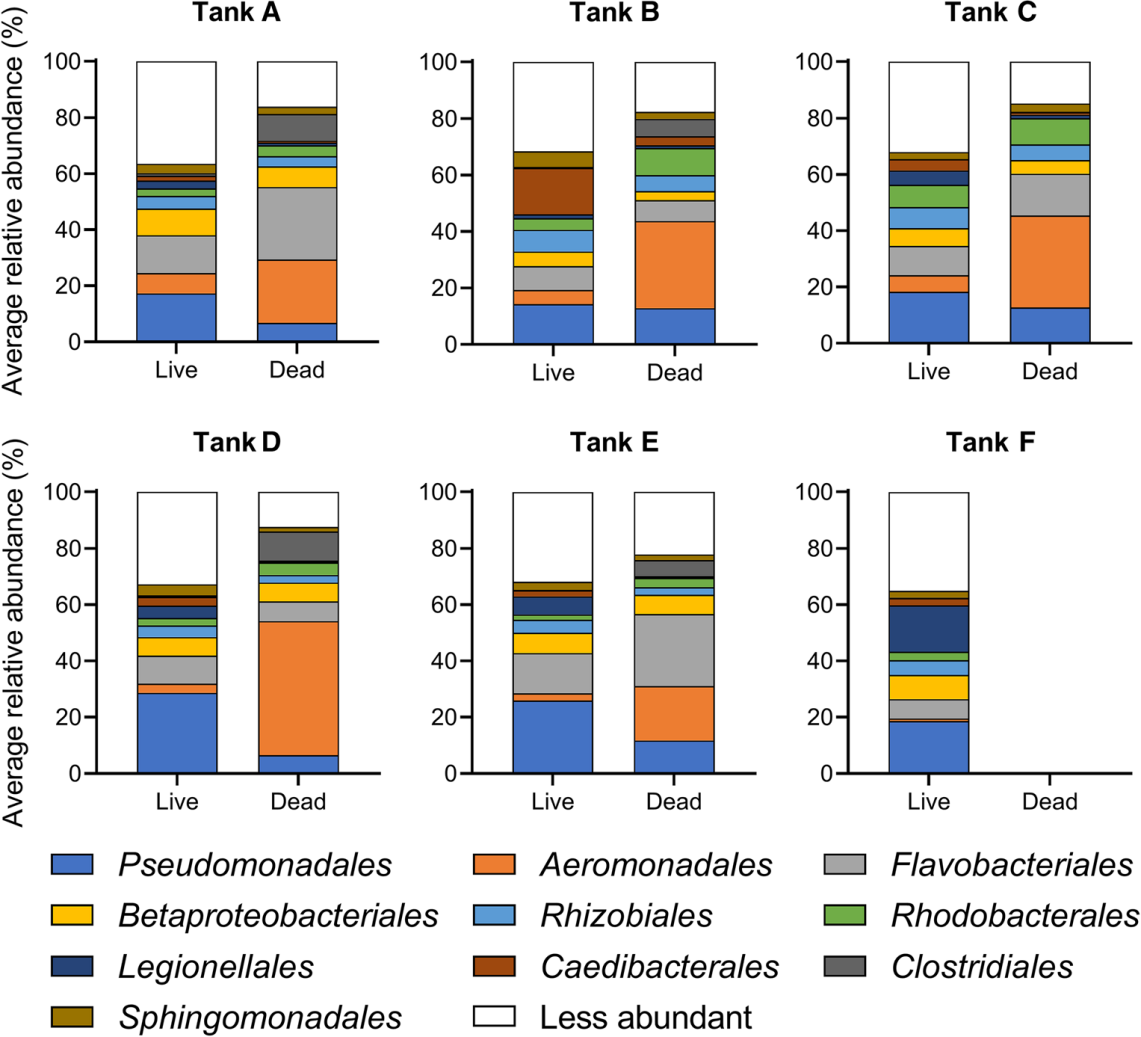
Order-level taxonomic classification of microbial communities associated with live and dead ZMs in experimental (A-E) and control tanks (F). All taxa present in ≥ 5% relative abundance in an individual ZM sample are shown in this graph

Order-level taxonomic analyses showed that live ZMs in the control tank (F) were dominated (% values) by *Pseudomonadales* (26.1 ± 22.6), *Legionellales* (23.3 ± 16.3), *Betaproteobacteriales* (12.1 ± 5.2), and *Flavobacteriales* (9.7 ± 9.2) (Fig. 3). Taxa that were enriched (≥ 3-fold) in dead ZMs within the experimental tanks included: *Aeromonadales* (tanks A-E), *Clostridiales* (tanks A, B, D, and E), *Flavobacteriales* (tanks A and E) and *Rhodobacterales* (tanks B and C); whereas those enriched in live ZMs within the experimental tanks included: *Aeromonadales* (tanks A, B, C, and D), *Clostridiales* (tank A), and *Caedibacterales* (tank B) (Fig. 3). Collectively, dead ZMs were mainly composed of *Aeromonadales* (29.4 ± 25.5), *Flavobacteriales* (16.7 ± 13.9), *Pseudomonadales* (10.6 ± 11.9), and *Rhodobacterales* (6.4 7 ± 7.7) (Fig. 3).

**Fig. 4:**
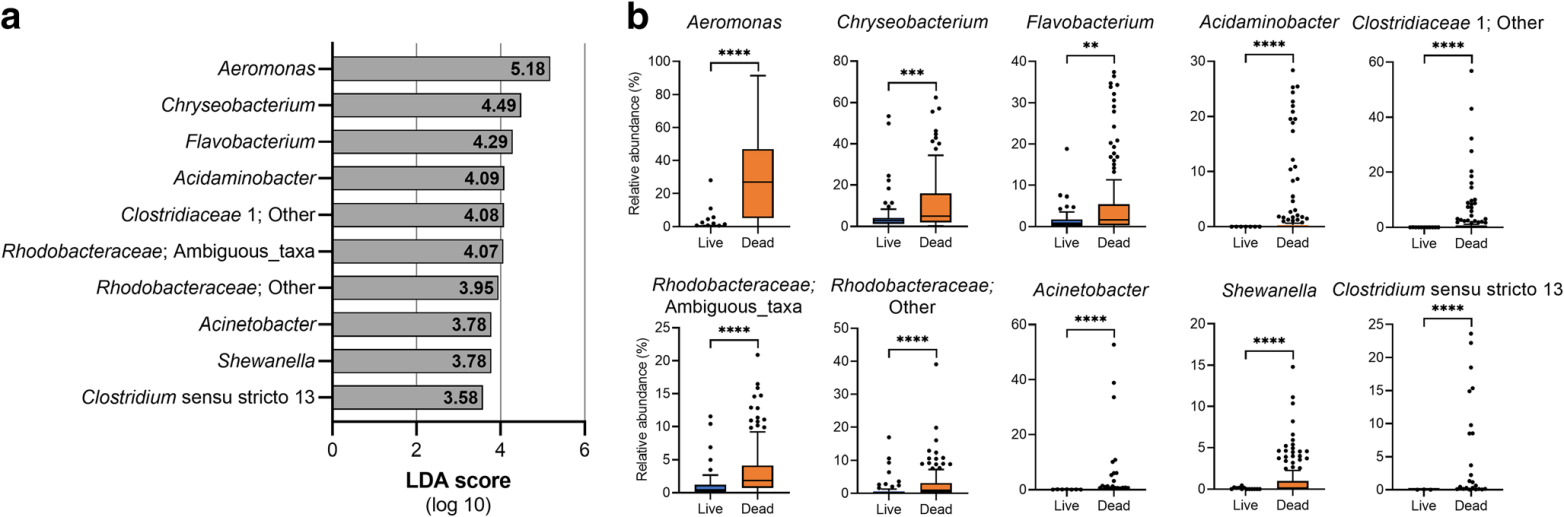
**a** Linear discriminant analysis effect size showing taxa that were significantly enriched in dead ZMs (from experimental tanks A-E) compared to live ZMs (from control tank F). Analysis was performed at an LDA cut-off score of 3.5. **b** Relative abundance of taxa that were significantly enriched in dead ZMs (from experimental tanks A-E) compared to live ZMs (from control tank F). *P* values ****≤ 0.0001, ***≤ 0.001, **≤ 0.01

The most abundant bacterial genera in live ZMs (control tank F) were *Pseudomonas* (20.0 ± 21.0), *Legionella* (7.7 ± 10.7), *Chryseobacterium* (7.7 ± 11.8), “*Ca.* Nucleicultrix” (3.9 ± 6.1), *Burkholderiaceae* (other) (3.8 ± 2.9), and *Aeromonas* (3.7 ± 10.7). In contrast, abundant genera in dead ZMs were comprised of *Aeromonas* (28.9 ± 25.3), *Chryseobacterium* (10.8 ± 12.9), *Pseudomonas* (9.4 ± 11.1), *Flavobacterium* (5.1 ± 8.5), and *Rhodobacteraceae* (ambiguous taxa) (3.3 ± 3.8). LEfSe analysis showed that members of the genera *Aeromonas*, *Chryseobacterium*, *Flavobacterium*, *Acidaminobacter*, *Clostridiaceae* 1 (other), *Rhodobacteraceae* (ambiguous taxa, other), *Acinetobacter*, *Shewanella*, and *Clostridium* sensu stricto 13 were significantly enriched (LDA score: ≥ 3.5) in dead ZMs (tanks A–E) compared to live ZMs (control tank F) (Fig. 4a, b). Redundancy analysis further confirmed that these enriched taxa, especially *Aeromonas*, were strongly associated with ZM mortality (Fig. S3).

Time-series analysis indicated a greater relative abundance of *Aeromonas* in dead ZMs compared to live ZMs from similar timepoints in each experimental tank (Fig. S4). Nevertheless, several live ZMs from the experimental tanks were also found to harbor *Aeromonas* at relative abundances similar to that of dead ZMs (Fig. S4). The relative abundance of *Aeromonas* in ZM feed and water samples was very low (0 to 0.29%), except for the water samples (0.84 to 5.04%) collected during the mortality events in tanks B and D on days 2 and 3 (Fig. S5).

### Proliferation of Putative Opportunistic Pathogens in Stressed ZMs

**Fig. 5:**
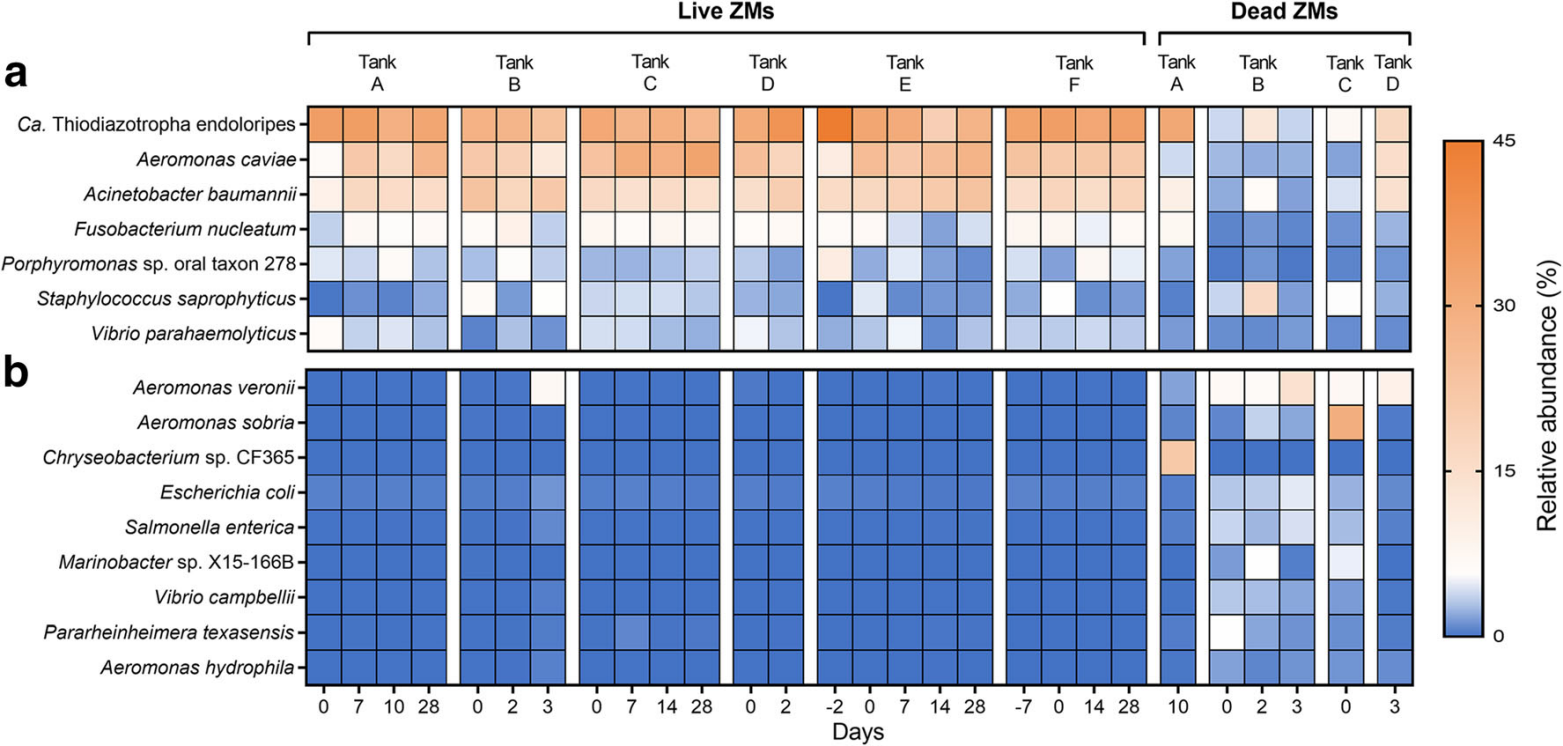
Shotgun sequence analysis of select live and dead ZMs from different tanks (**a**) Most abundant bacterial species (≥ 5% relative abundance) in live ZMs, and (**b**) Bacterial species that increased ≥5-fold in relative abundance in dead ZMs compared to live ZMs

Shotgun sequencing was performed on live and dead pooled ZMs from each tank to determine the species-level identity of microbial communities impacted by the temperature and salinity treatments. The three most abundant species in live ZMs were “*Candidatus* (*Ca.*) *Thiodiazotropha endoloripes*” (31.07 ± 4.98), *Aeromonas caviae* (21.84 ± 6.32), and *Acinetobacter baumannii* (17.17 ± 3.16) (Fig. 5a). In contrast, the relative abundance of all three species declined significantly (*p* ≤ 0.0001) in dead ZMs (Fig. 5a). Perhaps more importantly, however, several pathogenic *Aeromonas* species, including *A. veronii*, *A. sobria*, *A. hydrophila*, *A. allosaccharophila*, *A. salmonicidia*, *A. schuberti*, *A. media*, and *A. simiae* were significantly (p ≤ 0.0001) enriched in dead ZMs, compared to live mussels (Fig. 5b, Fig. S6). *Aeromonas veronii* was strongly associated with mortality induced by high temperature, whereas *A. sobria* was enriched in dead ZMs from the high salinity tank (tank C). Other species that were overrepresented in dead ZMs included: *Chryseobacterium* sp. CF365, *Escherichia coli*, *Salmonella enterica*, *Marinobacter* sp. X15-166B, *Vibrio campbellii*, and *Pararheinheimera texasensis* (Fig. 5b). Interestingly, live ZMs from one data point (tank B, day 3) displayed a microbiota profile similar to that of dead ZMs, which was in contrast to that seen in all other live ZMs (Fig. 5, Fig. S6).

**Fig. 6:**
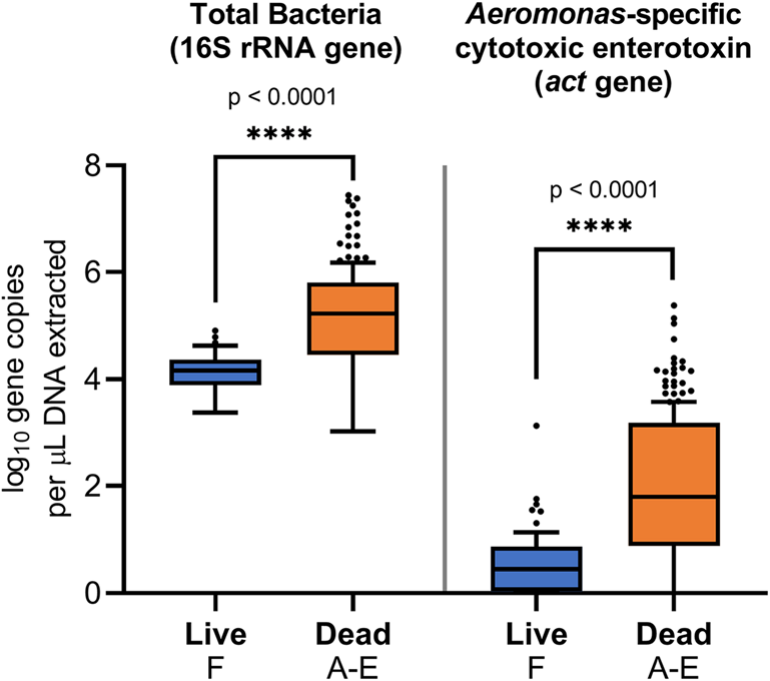
Quantification of 16S rRNA (total bacteria) and *act* (*Aeromonas*-specific cytotoxic enterotoxin) gene in dead ZMs (from experimental tanks A-E) and live ZMs (from control tank F). Tukey’s HSD post-hoc test. On the boxplots, the centerlines show the medians, the bottom and upper limits indicate the 25th and 75th percentiles and the whiskers encompass the 10–90 percentile range. *P* value ****≤ 0.0001

Quantitative PCR indicated that total bacterial abundance, based on the 16S rRNA gene, was significantly greater (*p* < 0.0001) in tissues of dead ZMs (Fig. 6). This also corresponded with a significant 322-fold increase (*p* < 0.0001) in the abundance of the cytotoxic enterotoxin (*act*) gene in dead ZMs, relative to live ZMs in the control tank (Fig. 6). Moreover, live ZMs from the experimental tanks (A–E) harbored a greater abundance of *act* gene copies compared to those from the control tank though this observation was not statistically significant (Fig. S7).

## Discussion

Results from this current study indicate that elevated temperature and salinity induced a shift in the ZM-associated microbiota, which may play an important role in its mortality. The mortality of ZMs in experimental tanks ranged between 53 and 100%, which was concomitant with significant increases in the relative abundance of putative opportunistic pathogenic bacteria in the genera *Aeromonas*, *Chryseobacterium*, and *Flavobacterium*. The potential involvement of aeromonads in ZM mortality was further supported by data showing that these microbes were present in much lower relative abundance in “healthy” ZMs from the control tank, where no mortality was observed. Shotgun sequencing and qPCR analyses revealed that the relative and absolute abundances of putative pathogenic *Aeromonas* species, particularly *A. veronii*, was significantly greater in dead ZMs and some live ZMs present in the experimental tanks than in those from control tanks. While these data are suggestive of the involvement of *Aeromonas* in ZM mortality, we are aware that they cannot reconcile issues of cause and effect. Nevertheless, these observations are relevant as the predicted increases in water temperature and/or salinity due to anthropogenic-induced climate changes have the potential to negatively impact these widespread invasive species, and as a consequence affect ecosystem services.

Temperature appeared to have a major impact on ZM mortality (Fig. 1, Fig. S3) as was also noted in a recent study [22]. While the authors concurred that individuals could not survive exposure to water temperatures greater than 33 °C [18], it was also reported that significant mortality was correlated with exposure time (hours) to temperatures > 25 °C. Similarly, our results show a clear correlation between exposure degree hours > 25 °C and mortality (Table S2). Both high temperature conditions examined (tanks B and D) resulted in severe and rapid mortality relative to what was seen in other tanks. The relatively higher mortality rate in tank D (high temperature) compared with tank B (high temperature + high salinity) suggests that mortality was somewhat modulated by conditioning to another stressor. While the temperature in the medium tank (tank E) was about half (9 °C vs. 15 °C) of that used in the high temperature tanks (B and D), the mortality was proportionally lower. Interestingly, it also appears that consistently high temperatures have a greater impact on ZM mortality compared to fluctuating temperatures, as the alternating temperature tank (A), which sees similarly high acute temperatures as the high temperature conditions does not have the same extent of mortality as did the high temperature conditions. Along similar lines, a recent study done using *Tigriopus californicus* reported that daily degree-hours best explained temperature wave induced mortality in this marine copepod [17].

Here, we also show that microbial diversity, as revealed by the Shannon index, was significantly reduced in dead ZMs from tanks B (high temperature + high salinity) and D (high temperature) relative to live ones in the control tank (Table 1). Moreover, dead ZMs were less diverse compared to live ZMs in each experimental tank (Table 1). Notably, a reduction in microbial diversity was also observed in some live ZMs collected from the experimental tanks prior to any mortality events (Fig. S2). It is a widely held concept among microbial ecologists that species diversity is strongly and positively correlated with ecosystem stability, and that high microbial diversity reduces perturbation-induced community decline or collapse [41, 42]. Lower microbial diversity has also been linked with impaired health status in bivalve species, including the Pacific oyster [43]. Furthermore, principal coordinates analysis revealed that microbial communities associated with dead ZMs in the experimental tanks clustered separately from those in live ZMs from the control tank (Fig. 2). Taken together, these results show that ZM mortality (or health status) is strongly associated with shifts in microbial community structure, although cause and effect as not been rigorously established.

Several mechanisms have been proposed by which stress-induced shifts in microbiota composition may deleteriously affect host fitness [44, 45]. For example, in humans and in advanced lower animals, it is well known that gut microbiota play a critical role in enhancing the digestive efficiency of complex food sources and in producing nutrients that are not host provided [46]. This is facilitated by microbial production of novel enzymes that the host is not capable of producing, or by increasing the abundance and (or) activity of enzymes critical to digestion [44, 45]. Hence, stress-induced alterations in gut microbiota may affect the metabolic costs and benefits received by the hosts. In addition to fulfilment of nutritional needs, a diverse and established microbiota also protects the host from invasion and colonization by pathogens via the secretion of antimicrobials, production of cell signaling molecules, the regulation of host genes, monopolization of resources, or by filling critical surface niches [44, 47, 48]. It is thought that disruption of the resident microbiota could reduce colonization resistance by opening up ecological niches in the gut for pathogens and/or by eliminating/reducing the abundances of gut bacteria that inhibit the growth of pathogens [45].

Members of the genus *Aeromonas* were strongly associated with dead ZMs (Fig. 4, Fig. S3). Subsequent species-level resolution data obtained by using shotgun sequencing indicated that *A. caviae* was the dominant *Aeromonas* species within live “healthy” ZMs. Moreover, ZM mortality was related to the decline of this bacterium, and a concomitant significant increase in the abundance of other *Aeromonas* species, especially *A. veronii* and *A. sobria* (Fig. 5, Fig. S6). A similar observation was also made with live ZMs collected from an experimental tank, suggesting that these mussels were on a trajectory towards death (Fig. S6). The contribution of *A. caviae* to host fitness is not known, but it appears that is a part of the microbial flora of healthy ZMs. These results also underscore the importance of examining microbial taxa at finer taxonomic resolution (species-level). Previous studies have demonstrated that activity of *A*. *media*, mediated by bacteriocins, was protective against several opportunistic pathogens of Pacific oysters, including other pathogenic *Aeromonas* strains [49, 50]. The authors later reported that antagonist activity in *A. media* was dependent on the production of indole (2,3 benzopyrrole) by the bacterium [51].

Our qPCR analyses indicated that there was a 322-fold increase in the abundance of the *act* gene in dead ZMs, relative to that found in live ZMs from the control tank, further confirming the proliferation of putative pathogenic *Aeromonas* species (Fig. 6). The *act* gene, which has been detected in *Aeromonas* isolates from fish [52] and mussels [53], encodes a secreted cytotoxic enterotoxin, which possesses hemolytic, cytotoxic, and enterotoxic activities. This toxin, which is among the most potent virulence factors in *Aeromonas* species, has been shown to induce lethality in a mouse model [54–56]. Our findings are in agreement with those from previous studies which demonstrated that *Aeromonas* spp. strain isolated from dead ZMs, such as *A. veronii*, *A. jandaei*, and *A. media*, were pathogenic to “healthy” live ZMs, and when re-inoculated resulted in mortality [57, 58], in many ways fulfilling Koch’s postulates

Lastly, several of these putative pathogens, including *Aeromonas*, were consistently detected in relatively low abundance in “non-stressed” ZMs from the control tank (Fig. 5, Fig. S4), suggesting that the natural microbiota in ZM likely act as the source of opportunistic pathogens that become less attenuated during stress events. More importantly, however, while the relative abundance of *Aeromonas* in the microalgae feed and tank water samples was very low, water and ZM tissue samples that were collected during mass mortality events in tanks B and D (Fig. S5) had exceedingly high concentrations of *Aeromonas spp.* strains. Based on these results, our data suggest that the likely source of these putative pathogens was the host’s natural microbiota and not those from an external environmental source.

Despite the strong association of *Aeromonas* spp. strain with dead ZMs, our study was not designed to separate out cause from effect. Establishment of this causal sequence must await more detailed microbial transplant studies and possibly the establishment of near axenic lines of ZMs. Moreover, we are acutely aware of the statistical limitations of this study, mainly imparted by space-induced limitations to sufficient replication in the BSL-2 invasive species containment facility. Due to this reason, readers should be cautious in trying to extend these results to the natural world—beyond this specific experiment.” That said, however, the very large differences in stressor-induced mortality in experimental tanks were quite obvious.

## Conclusions

Our findings strongly suggest that environmental stressors, especially elevated temperature, have the potential to induce ZM mortality. The observed changes within bacterial community structure and diversity in dead ZMs may be due to direct or indirect effects. Direct effects include stressor-induced changes to the growth rate of specific bacterial taxa, including putative pathogens. In contrast indirect effects may be due to stressor-induced decline of commensal bacteria, or to multiple synergistic effects on microbiota and host metabolic processes. Independent of cause, the negative effects of stressors on ZMs likely include increased relative growth rates of putative pathogens, reduced health benefits, and/or decreased immune function. Results of this study will be useful for understanding ZM biology, as well as the identification of abiotic and biotic factors that influence the fitness of this highly invasive bivalve species.

## Supplementary Information

ESM 1 (DOCX 1982 kb)
