# Supplementary material for: Influence of Environmental Stressors on the Microbiota of Zebra Mussels (Dreissena polymorpha)
Source: Microb Ecol. 2020 Nov 26;81(4):1042–53. doi: 10.1007/s00248-020-01642-2 (PMC8062372; doi:10.1007/s00248-020-01642-2)

**SUPPLEMENTAL FILE**

**Influence of Environmental Stressors on the Microbiota of Zebra Mussels (*Dreissena polymorpha*)**

Prince P. Mathai^1ǂ^, Jonathan H. Bertram^1ǂ^, Soumesh K. Padhi^2^, Vikash Singh^3^, Isaiah E. Tolo^2^, Alex Primus^3^, Sunil K. Mor^3^, Nicholas B.D. Phelps^2^, and Michael J. Sadowsky^1,4,5*^

^1^ BioTechnology Institute, University of Minnesota, St. Paul, MN, USA

^2^ Department of Fisheries, Wildlife, and Conservation Biology, University of Minnesota, St. Paul, MN, USA

^3^ Department of Veterinary Population Medicine, University of Minnesota, St. Paul, MN, USA

^4^ Department of Soil, Water, and Climate, University of Minnesota, St. Paul, MN, USA

^5^ Department of Plant and Microbial Biology, University of Minnesota, St. Paul, MN, USA

^ǂ^ These authors contributed equally to this work

*Correspondence:

Michael J. Sadowsky: 1479 Gortner Ave., 140 Gortner Labs, BioTechnology Institute, University of Minnesota, St. Paul, MN 55108 USA; Tel.: +1 (612) 624-2706; Fax: +1 (612) 625-5780; Email: sadowsky@umn.edu

**Table S1:** ZM mortality rates by tank

| **Tank** | **deg·hr>25 during acclimation** | **Days  until ΔR_mortality_** | **deg·hr>25 until ΔR_mortality_** | **Days  until L_D_=50%** | **deg·hr>25 until L_D_=50%** | **Final deg·hr>25** | **Average experimental mort. /day** |
| --- | --- | --- | --- | --- | --- | --- | --- |
| A | 100 | 8 | 450 | 11 | 500 | 1100 | 2.8 ± 4.7 |
| B | 400 | 2 | 600 | 2 | 650 | 1700 | 23.4 ± 17.4 |
| C | - | - | - | 16 | - | 0 | 1.1 ± 1.4 |
| D | 400 | 3 | 850 | 3 | 850 | 950 | 29.8 ± 58.3 |
| E | 25 | 11 | 400 | 17 | 600 | 1100 | 2.1 ± 2.2 |
| F | - | - | - | - | - | 0 | 0.0 ± 0.0 |

ΔR_mortality_ = mortality increased to greater than 2.5% of population; based on pre-trial observations

L_D_ = the point at which the treatment is lethal to a proportion of the population

*Degree hours were calculated from the start of the acclimation phase

- indicates not applicable

**Table S2:** Results of multivariate statistical tests based on the Bray–Curtis dissimilarities

| **#** | **Group** | **ANOSIM** | | **PERMANOVA** | | | **PERMDISP** | | |
| --- | --- | --- | --- | --- | --- | --- | --- | --- | --- |
|  |  | **R statistic** | **p-value** | **F.Model** | **R^2^** | **Pr(>F)** | **F** | **Pr(>F)** | **Pairwise p-value** |
| A | A (live)  A (dead)  F (live) | 0.3383 | 0.001 | 9.6069 | 0.15104 | 0.001 | 1.2271 | 0.287 | F (live) : A (dead) = 0.457  F (live) : A (live) = 0.314  A (dead) : A (live) = 0.166 |
| B | B (live)  B (dead)  F (live) | 0.4200 | 0.001 | 8.8364 | 0.18281 | 0.001 | 2.946 | 0.056 | F (live) : B (dead) = 0.909  F (live) : B (live) = 0.021  B (dead) : B (live) = 0.086 |
| C | C (live)  C (dead)  F (live) | 0.3940 | 0.001 | 10.6 | 0.18244 | 0.001 | 2.713 | 0.069 | F (live) : C (dead) = 0.076  F (live) : C (live) = 0.025  C (dead) : C (live) = 0.635 |
| D | D (live)  D (dead)  F (live) | 0.5017 | 0.001 | 8.8032 | 0.21843 | 0.001 | 7.3131 | 0.002 | F (live) : D (dead) = 0.001  F (live) : D (live) = 0.222  D (dead) : D (live) = 0.173 |
| E | E (live)  E (dead)  F (live) | 0.3716 | 0.001 | 9.6724 | 0.15311 | 0.001 | 0.4328 | 0.671 | F (live) : E (dead) = 0.746  F (live) : E (live) = 0.395  E (dead) : E (live) = 0.608 |

**Figure S1:** Monitoring water quality parameters throughout the experiment

**
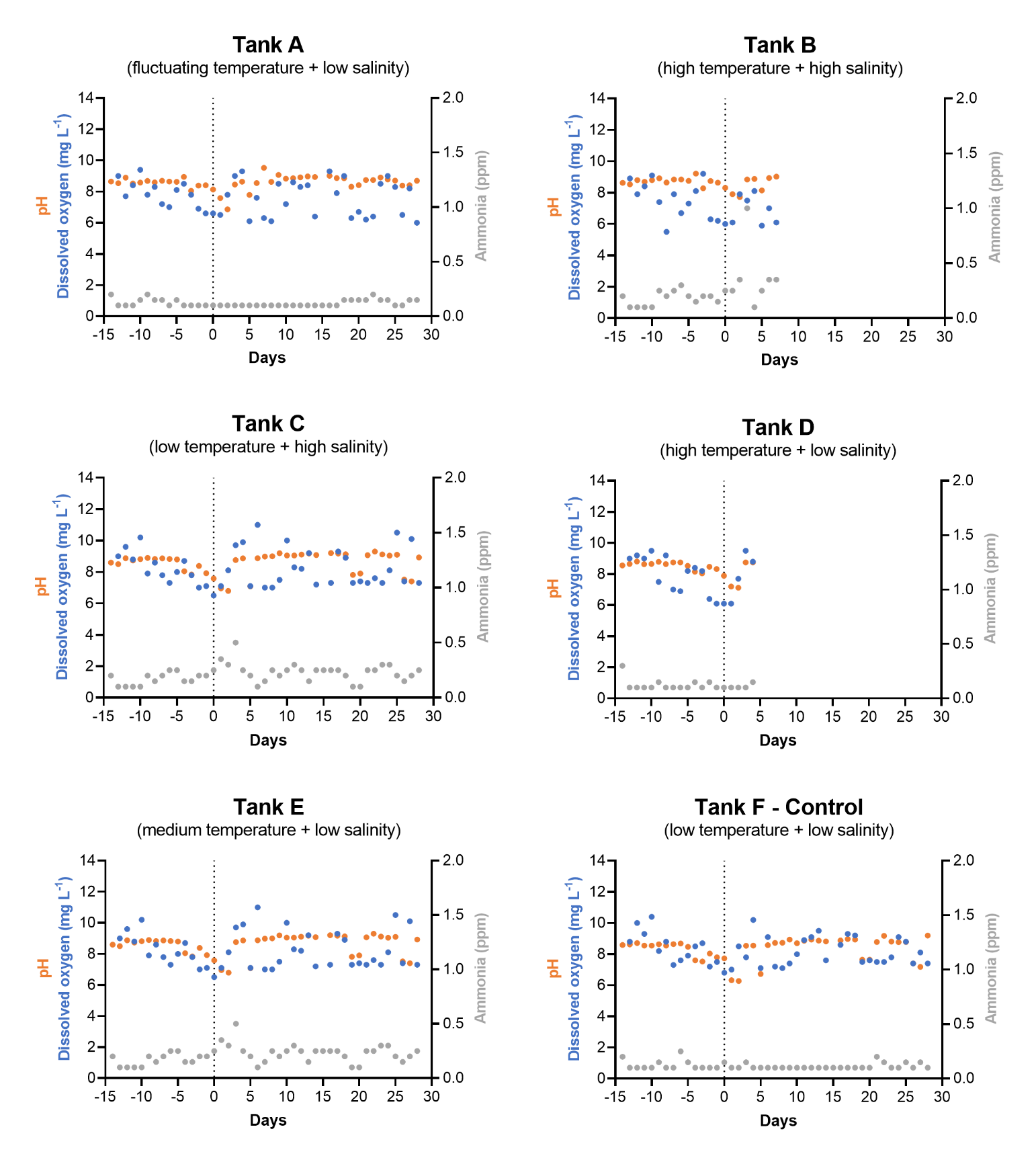
**

Dashed vertical line indicates end of acclimation phase (14 d) and start of experimental phase (28 d)

**Figure S2:** Time-series analysis depicting the diversity of microbial communities associated with live (blue) and dead (orange) ZMs in experimental (A-E) and control tanks (F)

**
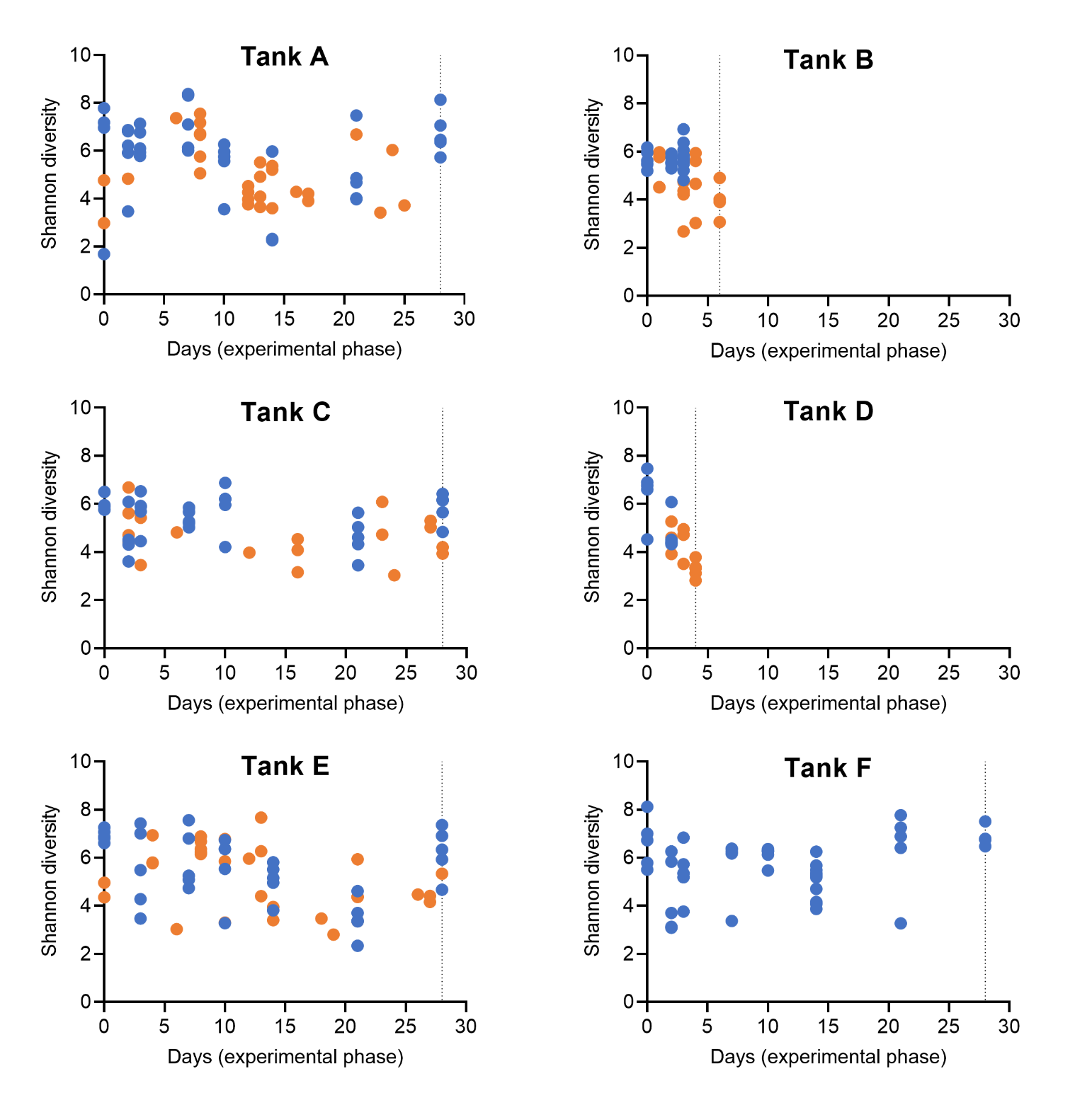
**

**Figure S3:** Redundancy analysis relating the relative abundance of select taxa with stressors (temperature/ salinity) and ZM health status (live/ dead)

**
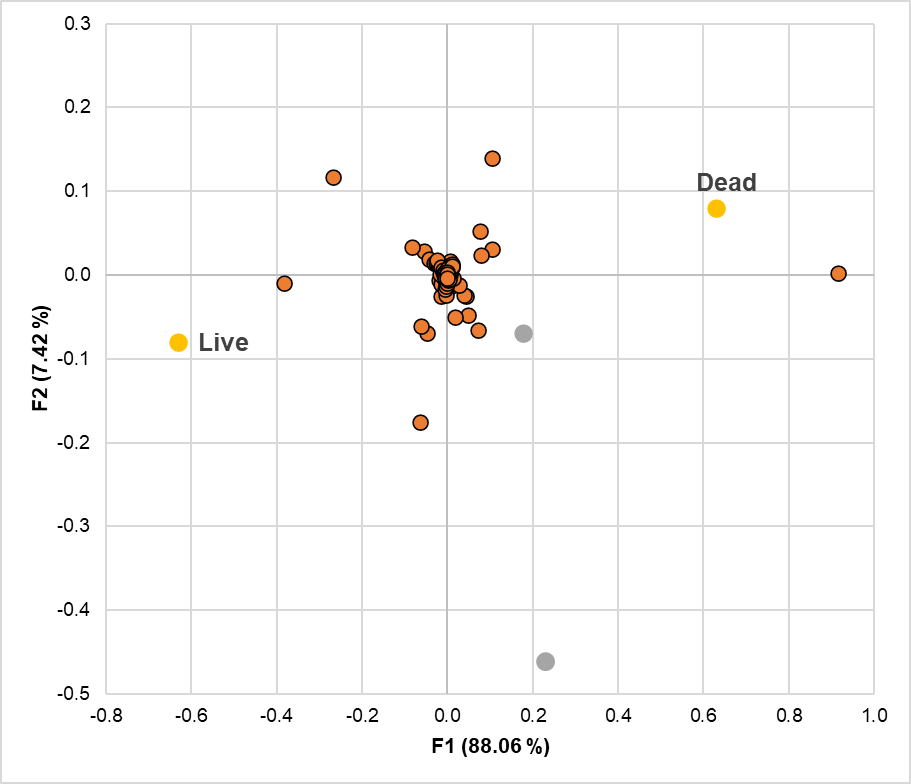
**

*Flavobacterium*

*Acidaminobacter*

*Clostridiaceae* 1

*Rhodobacteraceae*

*Ca.* Nucleicultrix

*Legionella*

*Chryseobacterium*

**Salinity**

**Temperature**

*Pseudomonas*

*Aeromonas*

**Figure S4:** Time-series analysis depicting the relative abundance of *Aeromonas* in live (blue) and dead (orange) ZMs in experimental (A-E) and control tanks (F)


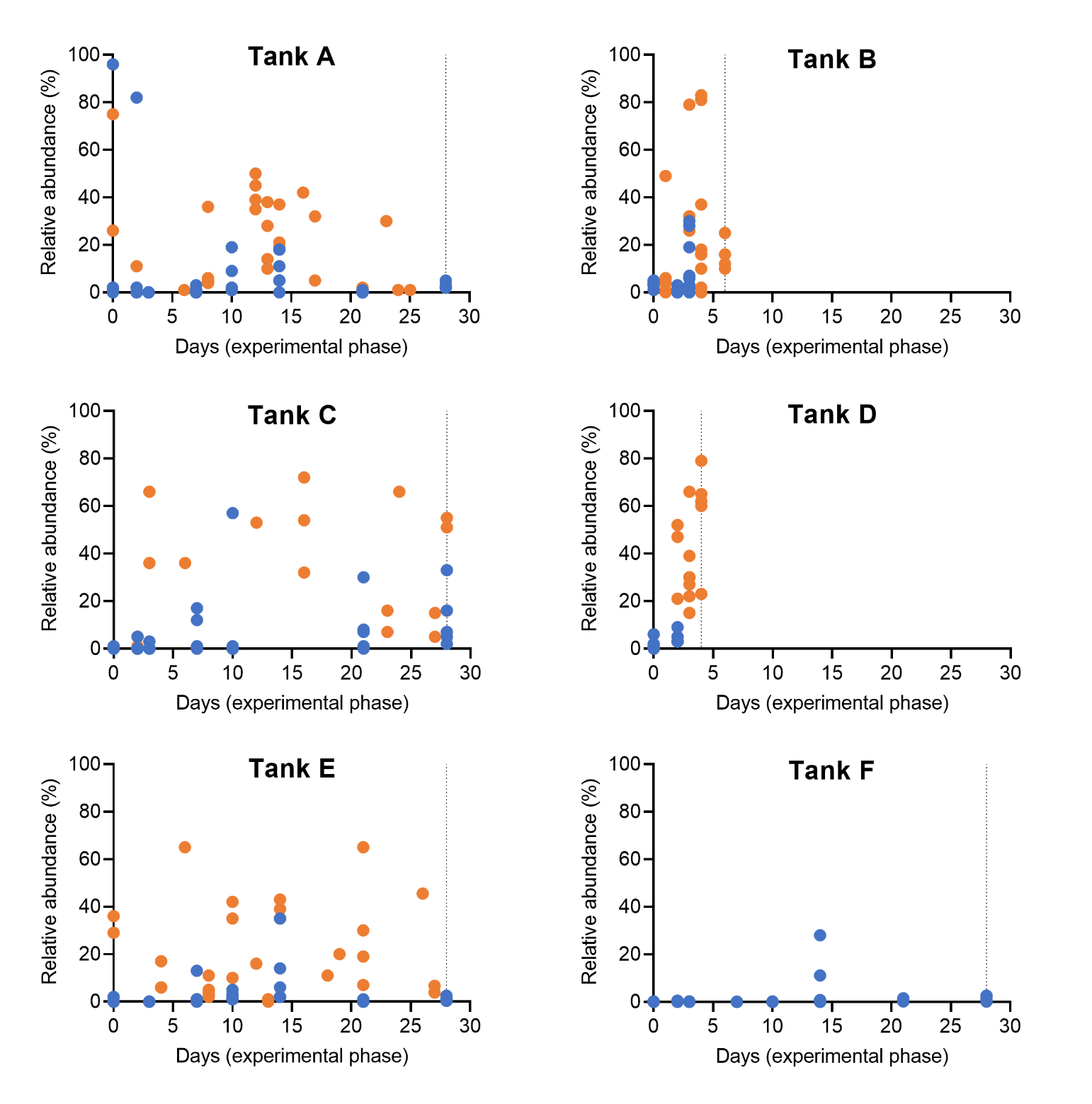


Dashed vertical lines indicate the end of the experimental phase in each respective tank.

**Figure S5:** Relative abundance of taxa (significantly enriched in dead ZMs) in ZM feed and water samples from experimental and control tanks


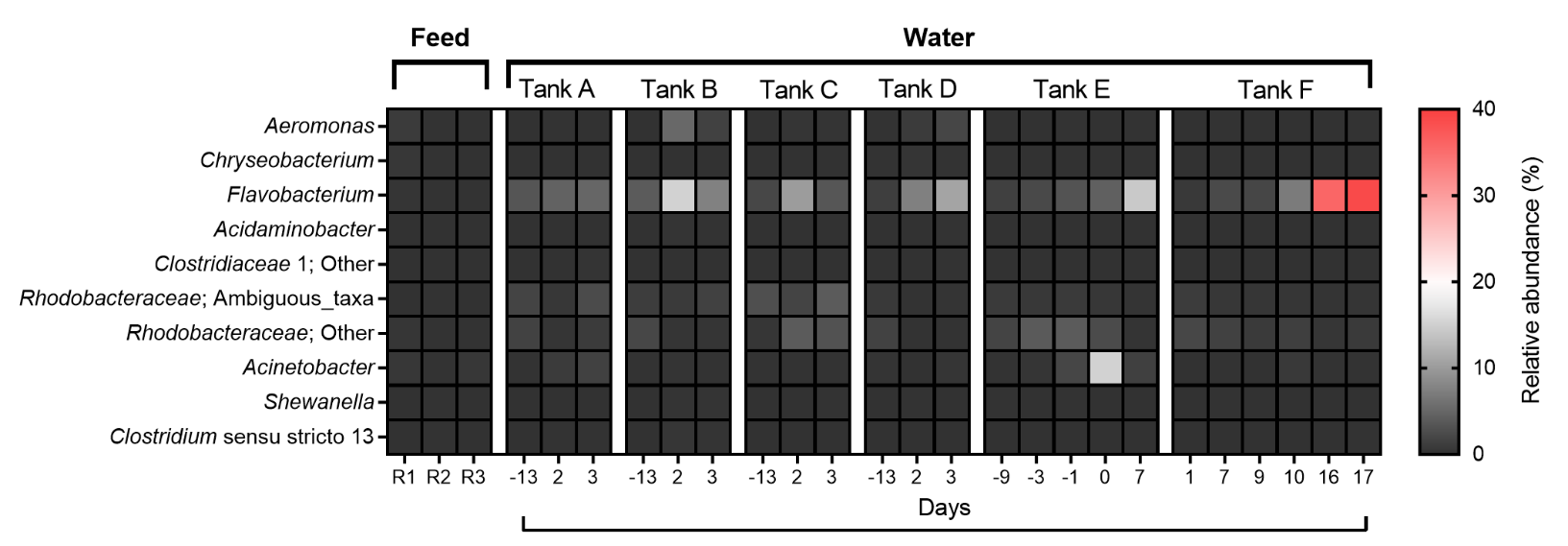


R1-3: indicate replicate samples

**Figure S6:** Shotgun sequence analysis showing relative abundance of *Aeromonas* species in live and dead ZMs


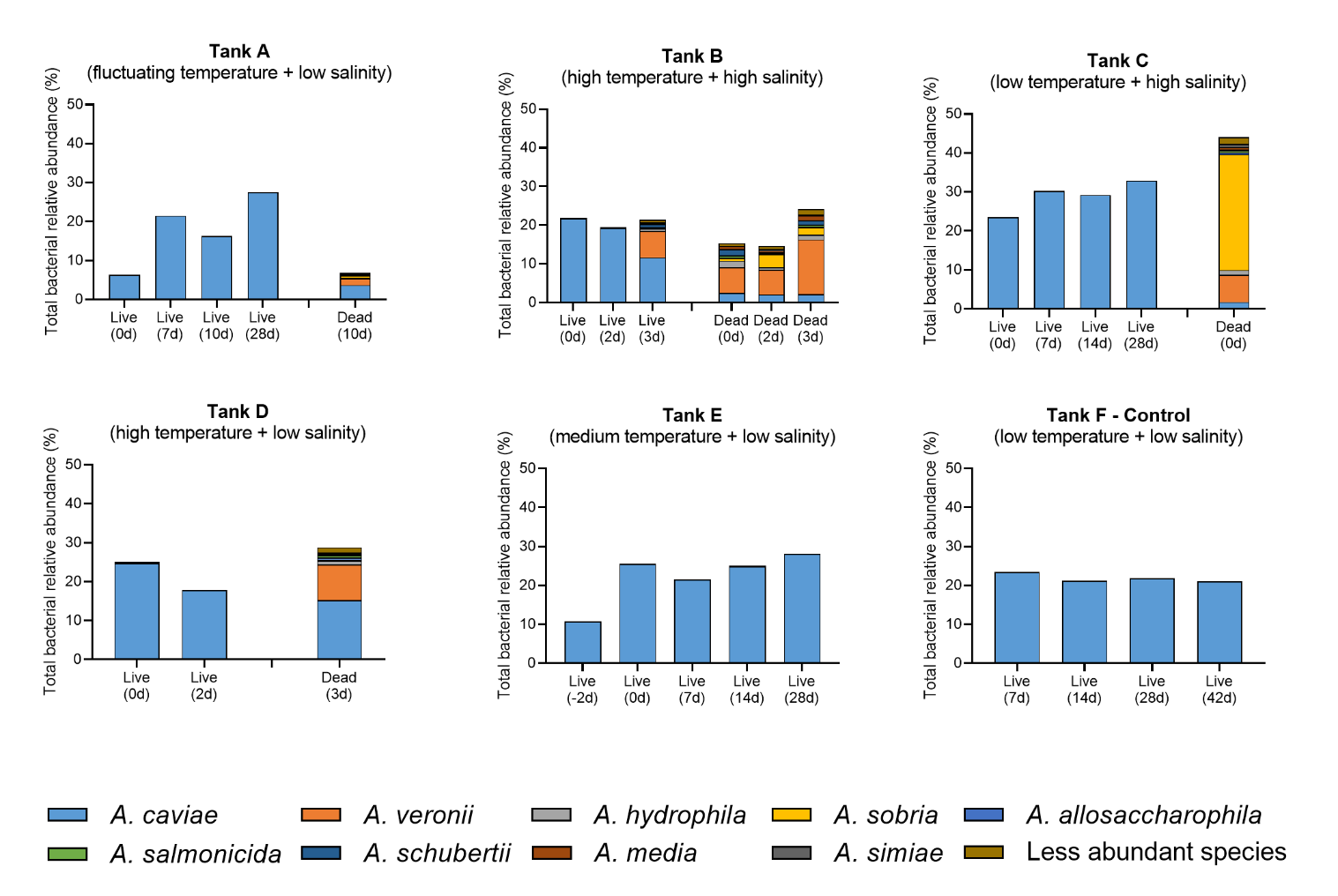


**Figure S7:** Quantification of the *Aeromonas*-specific cytotoxic enterotoxin (*act*) gene in live and dead ZMs from tanks A-F


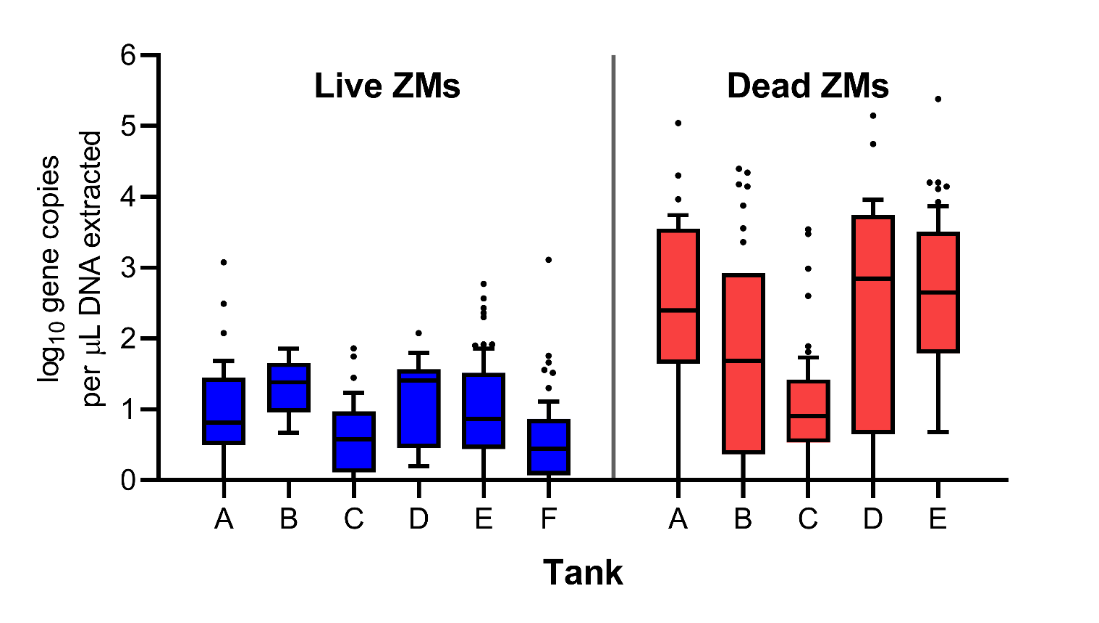

Supplement: Supplementary file 1 — (DOCX 1982 kb) [file 248_2020_1642_MOESM1_ESM.docx]
